# Cooperative function of oncogenic MAPK signaling and the loss of Pten for melanoma migration through the formation of lamellipodia

**DOI:** 10.1038/s41598-024-52020-8

**Published:** 2024-01-17

**Authors:** Yutaka Yasuta, Ryuya Kaminaka, Shutaro Nagai, Shuto Mouri, Katsuya Ishida, Akihiro Tanaka, Yue Zhou, Hiroaki Sakurai, Satoru Yokoyama

**Affiliations:** Department of Cancer Cell Biology, Faculty of Pharmaceutical Sciences, University of Toyama, 2630 Sugitani, Toyama, 930-0194 Japan

**Keywords:** Metastasis, Melanoma

## Abstract

The combination of oncogenes and tumor suppressors is involved in cancer development; however, it is still unknown whether their combination plays a critical role in cancer metastasis. We herein investigated whether genetic combinations affected cell migration ability by establishing the immortalized melanocytes, melan-a cells, with an oncogene, either BRAF^V600E^ or GNA11^Q209L^, and the loss of mouse Pten. The loss of mouse Pten or human PTEN increased the cell migration ability of our established cells and human melanoma cell lines with oncogenic MAPK signaling and the BRAF^V600E^ or NRAS^Q61R^ background, but not with the GNA11^Q209L^ background or no oncogenes. Although increased migration was not related to PI3K-AKT activation, those migration is regulated by the induction of some components in the WAVE regulatory complex, resulting in a higher rate of the formation of lamellipodia. On the other hand, BRAF^V600E^ induced EphA2 phosphorylation at serine 897 through RSK and was also required for cell migration and the formation of lamellipodia. Therefore, the oncogenic MAPK pathway and loss of Pten in melanoma were important for cell migration through the formation of lamellipodia, suggesting the significance of an appropriate combination of genetic alterations not only in cancer development, but also cancer metastasis.

## Introduction

The combination of genetic alterations in oncogenes or tumor suppressors is dependent on their tissue of origin and their genetic background of each cancer^1^. In cutaneous melanoma, the most common combination is the oncogene, BRAF^V600E^, with the loss of the tumor suppressor, PTEN^2^. In contrast, the oncogene, GNA11^Q209L^ or GNAQ^Q209L^, with the loss of the tumor suppressor, BAP1, is mainly reported in uveal melanoma^3,4^. Since evolutionary selective pressure occurs during melanomagenesis, genetic combinations are considered to play a functional role in cancer development or survival; however, it is still unknown whether these genetic combinations are involved in cancer metastasis. A more detailed clinical understanding of genetic combinations will provide insights into synthetic lethality and synthetic essentiality^5–7^.

Among melanoma-related genes, the loss of PTEN is a significant event during the development of melanoma and has been correlated with its metastasis in human clinical specimens and genetically engineered mouse models^8–10^. The loss of PTEN frequently co-occurs with BRAF mutations^11,12^. An increase in brain metastatic ability through the combination of oncogenic BRAF and the loss of PTEN has been suggested epidemiologically^9^; however, the mechanisms by which the loss of PTEN in melanoma with mutations in the BRAF gene induce metastasis have not yet been elucidated.

Cancer metastasis consists of several steps: intravasation, attachment to a vessel, extravasation, angiogenesis, and growth in distal tissues^13^. The migration ability or invasiveness of cancer cells plays a critical role during metastasis. Directional migration involves the protrusion of the leading edge of a cell through the formation of filopodia or lamellipodia, which comprise actin filaments. Since cancer metastasis involves oncogenes and tumor suppressors, they may regulate the formation of filopodia or lamellipodia. The oncogene RAC1 in melanoma is a member of the Rho family of GTPases^14,15^, and activates the WAVE regulatory complex (WRC). WRC includes a number of components, such as Arp2/3, WAVE1/2, Abi1, and CYFIP1, and plays a role in the reorganization of actin and formation of lamellipodia^16^.

In the present study, we established mouse melanocyte lines with oncogenes, either BRAF^V600E^ or GNA11^Q209L^, or knocked out the tumor suppressor Pten from the mouse immortalized melanocyte cell line, melan-a. Although the growth rate of these cells in vitro remained unchanged, BRAF^V600E^/Pten^−/−^-melan-a cells showed a significantly higher migration ability with the formation of lamellipodia than other series of melan-a cells. Furthermore, cells lacking Pten expressed higher levels of some components of WRC. An inhibitor of Arp2/3, a component of WRC, significantly suppressed cell migration by BRAF^V600E^/Pten^−/−^-melan-a cells. In addition to the regulation of WRC by Pten^−/−^, the migration of BRAF^V600E^/Pten^−/−^-melan-a cells was suppressed by inhibitors of RSK, a downstream serine/threonine kinase of oncogenic BRAF^V600E^ that phosphorylates EphA2 at serine 897^17^. Moreover, the EphA2 inhibitor, ALW II-41-27, suppressed cell migration and the formation of lamellipodia in concert with a reduction in the serine 897 phosphorylation of EphA2. These results suggest that the increased migration ability of BRAF^V600E^/Pten^−/−^-cells was dependent on the formation of lamellipodia through the regulation of WRC by Pten^−/−^ as well as EphA2 serine phosphorylation by the BRAF^V600E^-RSK axis.

## Results

### Establishment of melan-a cells overexpressing oncogenes or with the knockout of Pten

**Figure 1:**
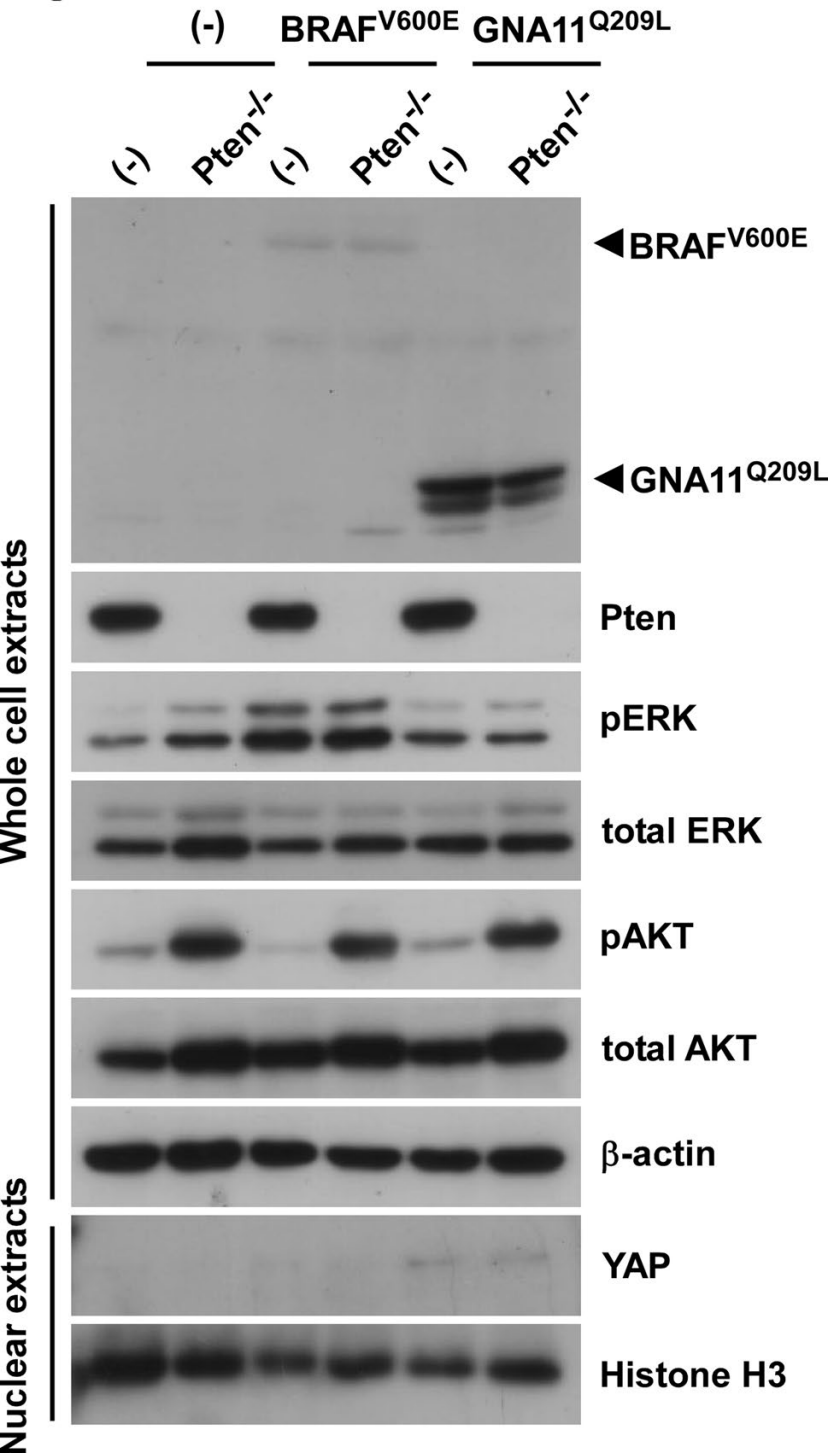
Establishment of a series of melan-a cells with oncogenes and the loss of Pten. Whole-cell lysates or nuclear extracts from six established melan-a cells: melan-a, Pten^−/−^-melan-a, BRAF^V600E^-melan-a, BRAF^V600E^/Pten^−/−^-melan-a, GNA11^Q209L^-melan-a, and GNA11^Q209L^/Pten^−/−^-melan-a, were subjected to Western blotting.

To examine the effects of genetic combinations in melanoma, we established the following mouse melanocyte lines from melan-a/Cas9 cells overexpressing spCas9: BRAF^V600E^-melan-a or GNA11^Q209L^-melan-a cells stably expressing BRAF^V600E^ or GNA11^Q209L^, which are oncogenic mutations in cutaneous or uveal melanoma, respectively. After single cell cloning, Pten was further knocked out using CRISPR-Cas9 systems (Pten^−/−^-melan-a, BRAF^V600E^/Pten^−/−^-melan-a, and GNA11^Q209L^/Pten^−/−^-melan-a) (Fig. 1). The expression of BRAF^V600E^ and loss of Pten were functionally confirmed by phosphorylated ERK or AKT, respectively, in concert with the overexpression of BRAF and loss of Pten. We also confirmed the nuclear localization of YAP1 by the overexpression of GNA11^Q209L^, which is regulated by active GNA11^18^. Although GNA11^Q209L^ activated ERK pathways similar as previous reports^19^, the phosphorylation levels of ERK was weaker than that by BRAF^V600E^. These results confirmed the establishment of a series of melan-a cell lines with BRAF^V600E^, GNA11^Q209L^, or the loss of Pten.

### The loss of Pten induces melanoma migration dependent on oncogenic MAPK activation

**Figure 2:**
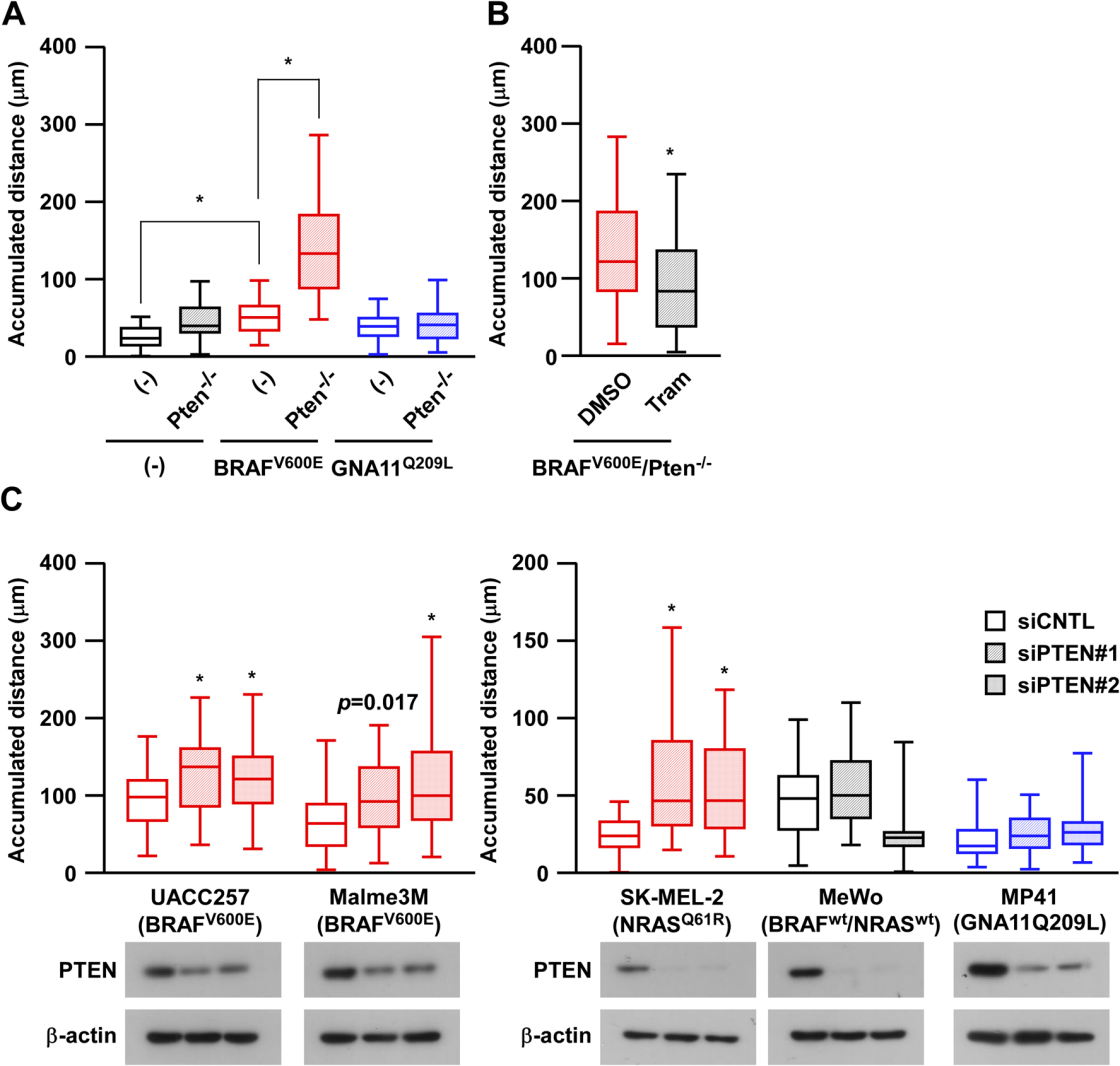
The loss of Pten induces melanoma migration in a manner that is dependent on oncogenic MAPK activation. (**A**) Cell migration in established melan-a cells was observed using the time-lapse imaging system for 3 h. Accumulated migrating distances are shown in box and whisker plots. *p < 0.01, by a one-way ANOVA followed by the Bonferroni post hoc test. (**B**) BRAF^V600E^/Pten^−/−^-melan-a cells were pre-treated with 30 nM trametinib for 24 h and cell migration in established melan-a cells was observed using the time-lapse imaging system for 3 h. *p < 0.01, using the unpaired Student’s *t*-test. (**C**) The human melanoma cell lines, UACC257, Malme3M, SK-MEL-2, MeWo, and MP41 were transfected with siCNTL or siPTEN (#1 and #2) and cell migration was observed using the time-lapse imaging system for 3 h. Other conditions were similar to those in (**A**).

In our established melan-a cells, we investigated the effects of the loss of Pten on cell growth (Supplementary Fig. S1A) and cell migration (Fig. 2A). Because melan-a cells are the immortalized cell lines^20^ and their growth are supported by addition of fetal bovine serum, in vitro growth abilities under normal growth condition, which contains fetal bovine serum, were not strongly affected by genetic alterations. Instead, the loss of Pten clearly induced cell migration under the BRAF^V600E^ background, but not under the GNA11^Q209L^ background or in the absence of oncogenes. Similar to Pten-knockout cells, the knockdown of Pten using siRNAs induced the migration of BRAF^V600E^-melan-a cells, but not wild-type or GNA11^Q209L^-melan-a cells (Supplementary Fig. S1B). In addition, the increased migration of BRAF^V600E^/Pten^−/−^-melan-a cells was suppressed by trametinib, a MEK inhibitor (Fig. 2B), suggesting a significant role for the activated MAPK pathway in Pten loss-induced migration.

We also examined the migratory effects of the loss of PTEN in human melanoma cells with wild-type PTEN, such as UACC257 and Malme3M cells with the BRAF^V600E^ mutation, SK-MEL-2 cells with the NRAS^Q61R^ mutation, MeWo cells with neither the BRAF, NRAS, nor NF1 mutation, and MP41 cells with the GNA11^Q209L^ mutation. Although the increase in migration observed in Malme3M cells with the knockdown of PTEN did not reach significance, increased migration ability after the knockdown of PTEN was observed in the cell lines with BRAF^V600E^ or NRAS^Q61R^, which constitutively activates the MAPK pathway, but not in MeWo cells (Fig. 2C). Similar to GNA11^Q209L^-melan-a cells, the knockdown of PTEN failed to induce the migration of MP41 uveal melanoma cells, suggesting that the increased migratory ability by the loss of PTEN was dependent on the oncogenic MAPK pathway.

#### The formation of lamellipodia-like structure is induced in BRAF^V600E^/Pten^−/−^-melan-a cells

**Figure 3:**
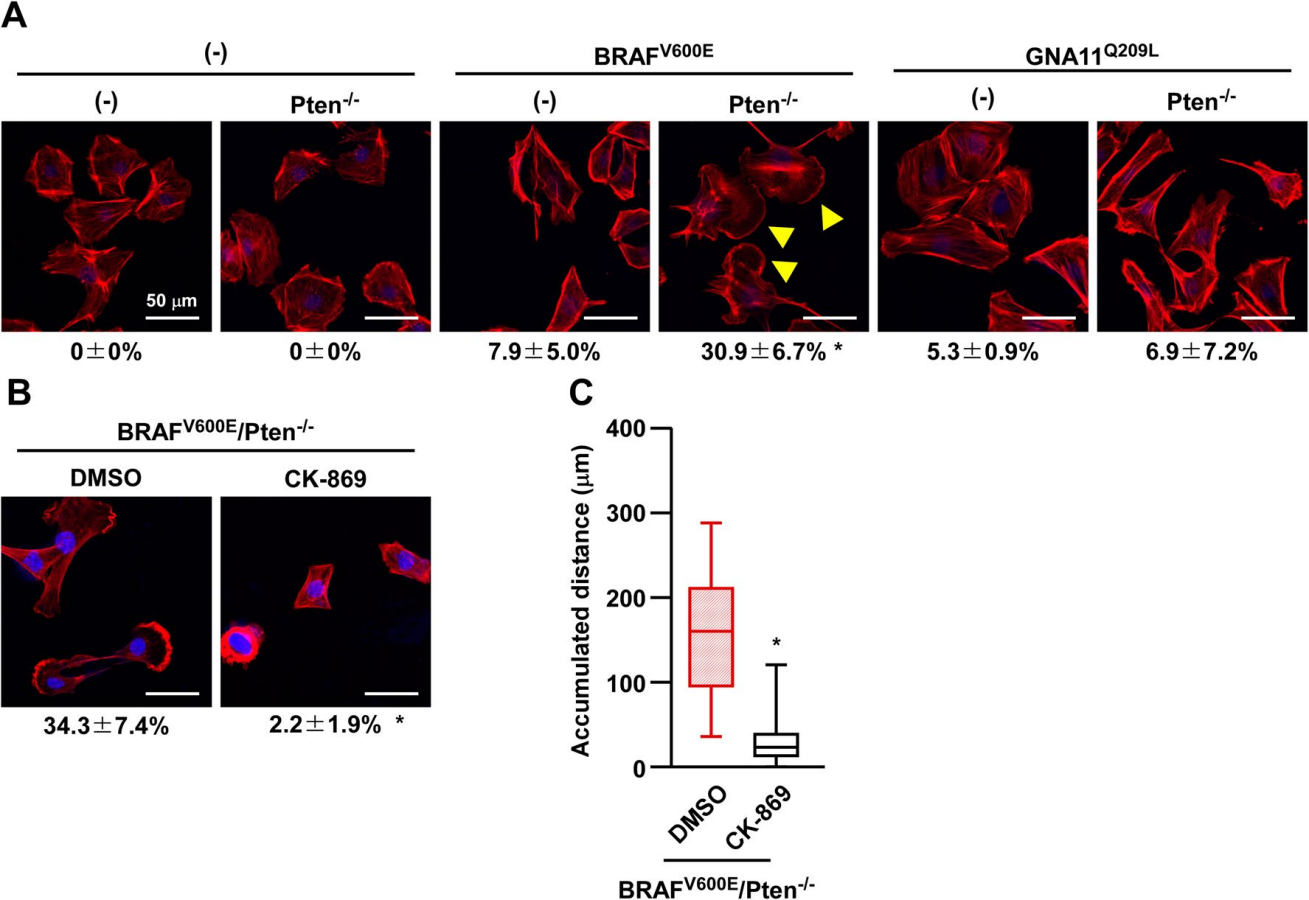
Lamellipodia-like structure is induced in BRAF^V600E^/Pten^−/−^-melan-a cells. (**A**) A series of melan-a cells were stained with rhodamine-conjugated phalloidin and DAPI. Images were recorded using a fluorescent microscope. Scale bar is 50 µm. Cells with lamellipodia-like structure (arrowhead) were counted and percentages are shown below images. Data are presented as the mean ± SD of at least three independent experiments. *p < 0.01 versus each corresponding non-Pten^−/−^ cells by two-way ANOVA followed by the Bonferroni’s post test. (**B**) BRAF^V600E^/Pten^−/−^-melan-a cells were pre-treated with 50 nM CK-869 for 1 h and then stained with rhodamine-conjugated phalloidin and DAPI. *p < 0.01 by student’s t-test. Other conditions were similar to those in (**A**). (**C**) BRAF^V600E^/Pten^−/−^-melan-a cells were pre-treated with 50 nM CK-869 for 24 h and cell migration was then observed using the time-lapse imaging system for 3 h. Other conditions were similar to those in Fig. 2A.

Since increases in migration ability are accompanied by morphological changes, we investigated the formation of lamellipodia-like structure by phalloidin staining, a membrane structure involved in cell migration. As shown in Fig. 3A, lamellipodia-like structure were detected in one third of BRAF^V600E^/Pten^−/−^-melan-a cells, but in less of the other melan-a cells. To establish whether the formation of lamellipodia-like structure contributed to the increases observed in cell migration, BRAF^V600E^/Pten^−/−^-melan-a cells were treated with CK-869, an inhibitor of Arp2/3, which is an essential molecule in the formation of lamellipodia via actin polymerization. Consistent with the loss of formation of lamellipodia-like structure after the CK-869 treatment (Fig. 3B), cell migration was completely inhibited by the CK-869 treatment in BRAF^V600E^/Pten^−/−^-melan-a cells (Fig. 3C), suggesting the significance of the formation of lamellipodia-like structures in the increases observed in the migration of BRAF^V600E^/Pten^−/−^-melan-a cells.

#### Abi1 is required for the Pten loss-induced migration of BRAF^V600E^/Pten^−/−^-melan-a cells

Although increases in cell migration by the loss of Pten or knockdown of PTEN were observed in cells with an oncogenic activated MAPK pathway (Fig. 2), there is currently no evidence to show that oncogenic BRAF with the loss of Pten coordinately regulates cell migration. The loss of Pten itself is known to regulate cell migration through the induction of epithelial-to-mesenchymal transition (EMT)^21^. However, we did not detect the induction of N-cadherin or SLUG expression, EMT markers, in our series of melan-a cells (Supplementary Fig. S2A). Furthermore, the PI3K inhibitor LY294002 and the AKT inhibitor MK2206 did not attenuate increases in cell migration (Supplementary Fig. S2B), although the loss of Pten activated the PI3K-AKT pathway (Fig. 1). Since the PI3K-AKT pathway is related to EMT with the loss of Pten, these results imply that the loss of the lipid phosphatase activity of Pten is an unlikely mechanism increasing the migration of BRAF^V600E^/Pten^−/−^-melan-a cells.

**Figure 4:**
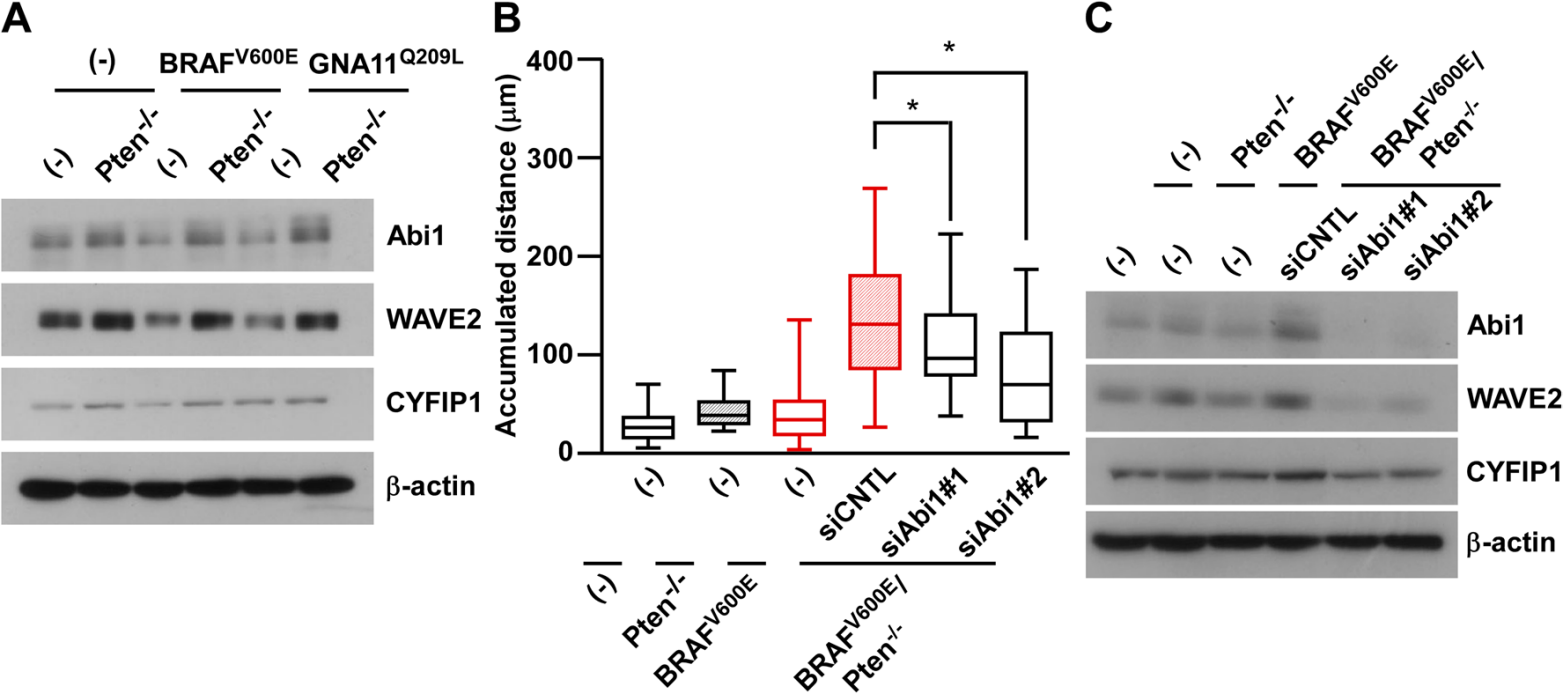
Abi1 is required for Pten^−/−^-induced cell migration in BRAF^V600E^/Pten^−/−^-melan-a cells. (**A**) Whole-cell lysates from six established melan-a cells: melan-a, Pten^−/−^-melan-a, BRAF^V600E^-melan-a, BRAF^V600E^/Pten^−/−^-melan-a, GNA11^Q209L^-melan-a, and GNA11^Q209L^/Pten^−/−^-melan-a, were subjected to Western blotting. (**B**, **C**) BRAF^V600E^/Pten^−/−^-melan-a cells were transfected with siRNAs for Abi1 (#1 or #2). After 96 h, cell migration was observed using the time-lapse imaging system for 3 h (**B**) or whole-cell lysates were subjected to Western blotting (**C**). Other conditions were similar to those in Fig. 2A.

Through its protein phosphatase activity, Pten dephosphorylates the Abi1 protein^22,23^, a component of WRC, which regulates the reorganization of actin and formation of lamellipodia^16^. Therefore, we examined the expression of Abi1 in established melan-a cells. As shown in Fig. 4A, the expression of the Abi1 protein was higher in a series of melan-a cells with the loss of Pten than in each counterpart expressing Pten. Consistent with previous findings^24–26^, other subunits of WRC, WAVE2 and CYFIP1, were also stabilized by the loss of Pten. To investigate the significance of Abi1 in the migration ability of BRAF^V600E^/Pten^−/−^-melan-a cells, Abi1 was knocked down by siRNA transfection. Cell migration ability significantly decreased after the knockdown of Abi1 (Fig. 4B), in concert with reductions in the expression of WAVE2 and CYFIP1 (Fig. 4C). These results suggest that the induction of Abi1, which may occur through the loss of the protein phosphatase activity of Pten, is essential for inducing the migration of BRAF^V600E^/Pten^−/−^-melan-a cells.

### BRAF-RSK-EphA2 regulates melanoma migration through the formation of lamellipodia

**Figure 5:**
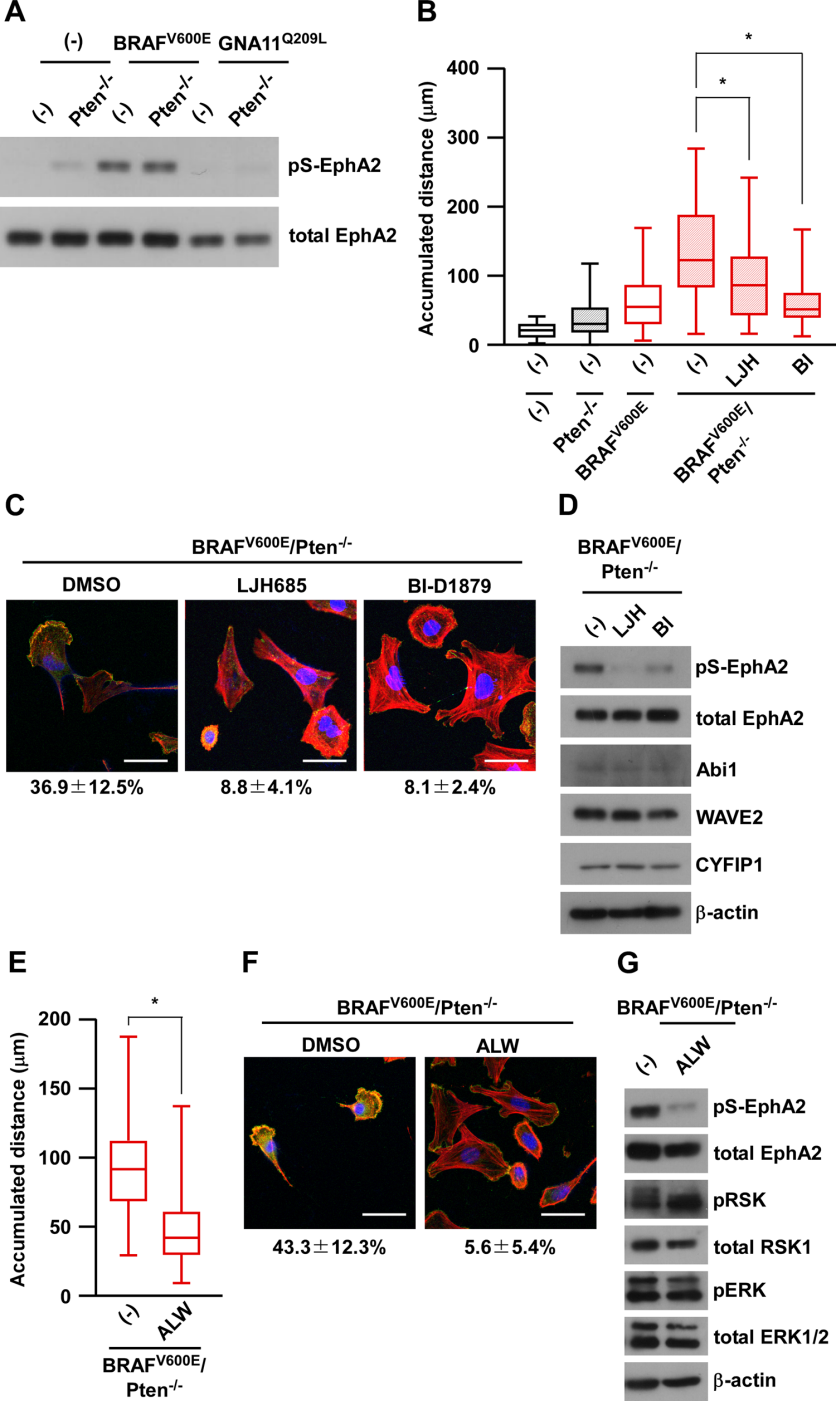
BRAF-RSK-EphA2 regulates melanoma migration through lamellipodia formation. (**A**) Whole-cell lysates from six established melan-a cells: melan-a, Pten^−/−^-melan-a, BRAF^V600E^-melan-a, BRAF^V600E^/Pten^−/−^-melan-a, GNA11^Q209L^-melan-a, and GNA11^Q209L^/Pten^−/−^-melan-a, were subjected to Western blotting. (**B**) BRAF^V600E^/Pten^−/−^-melan-a cells were pre-treated with RSK inhibitors, 50 μM LJH589 (LJH) or 10 μM BI-D1879 (BI), for 24 h and cell migration was observed using the time-lapse imaging system for 3 h. (**C**, **D**) BRAF^V600E^/Pten^−/−^-melan-a cells were pre-treated with RSK inhibitors, 50 μM LJH589 (LJH) or 10 μM BI-D1879 (BI), for 1 h and then stained with WAVE2 (green), rhodamine-conjugated phalloidin (red), and DAPI (blue) (**C**), or whole-cell lysates were subjected to Western blotting (**D**). Scale bar is 50 µm. The cells with lamellipodia structure (WAVE2-positive) were counted and percentages are shown below images. Data are presented as the mean ± SD of at least three independent experiments. *p < 0.01 versus DMSO-treated cells by one-way ANOVA followed by the Bonferroni’s post test. (**E**–**G**) BRAF^V600E^/Pten^−/−^-melan-a cells were pre-treated with the EphA2 inhibitor, 250 nM ALW II-41–27 (ALW) for 24 h and cell migration was observed using the time-lapse imaging system for 3 h (**E**). After the ALW treatment, cells were stained with WAVE2 (green), rhodamine-conjugated phalloidin (red), and DAPI (blue) (**F**), or whole-cell lysates were subjected to Western blotting (**G**). *p < 0.01 by student’s t-test.

RSK, a kinase downstream of BRAF, regulates cell migration through the phosphorylation of EphA2 at serine 897, which preferentially localizes to lamellipodia^17^. To confirm the involvement of EphA2 in cell migration, we examined the serine 897 phosphorylation of EphA2 (pS-EphA2) in a series of melan-a cells (Fig. 5A). As shown in Fig. 5A, serine 897 phosphorylation was induced downstream of the BRAF^V600E^ mutation. Due to faint ERK activation in GNA11^Q209L^-melan-a cells (Fig. 1), pS-EphA2 was not induced, suggesting the insufficient MAPK activation in GNA11^Q209L^. In addition, the migration ability by BRAF^V600E^/Pten^−/−^-melan-a cells was significantly inhibited by RSK inhibitors (Fig. 5B). To further confirm the lamellipodia formation, WAVE2, which is essential for lamellipodia formation, was co-stained with actin structures. Shown in Fig. 5C, WAVE2 was localized to the membrane in BRAF^V600E^/Pten^−/−^-melan-a cells, suggesting the lamellipodia formation. Although WAVE2 expression is increased (Fig. 4A), diffused staining of WAVE2 in cytoplasm was observed in Pten^−/−^-melan-a cells (Supplemental Fig. S3A). Moreover, pS-EphA2 as well as the formation of lamellipodia in BRAF^V600E^/Pten^−/−^-melan-a cells were reduced after the treatment with RSK inhibitors, though the expression of some WRC components, namely, WAVE2, CYFIP1, and Abi1, was not affected, even in cells treated with RSK inhibitors (Fig. 5D). We also confirmed the reduction of pS-EphA2 by RSK inhibitor in human melanoma cell lines with BRAF^V600E^ or NRAS^Q61R^ mutation, but not in other cell lines (Supplemental Fig. S3B). These results suggest that the increase in migration induced by the RSK-EphA2 axis may be independent of the expression of WRC components. We also examined the effects of the EphA2 inhibitor, ALW II-41-27 (ALW)^27^, on cell migration. Similar to RSK inhibitors, ALW inhibited cell migration (Fig. 5E), in concert with the suppressed formation of lamellipodia (Fig. 5F) and pS-EphA2 (Fig. 5G), in BRAF^V600E^/Pten^−/−^-melan-a cells. These results clearly suggest the critical role of the RSK-pS-EphA2 axis in the formation of lamellipodia and in an increase in the migration of melanoma.

## Discussion

We herein demonstrated that migration ability was increased by the combination of BRAF^V600E^ with the loss of Pten, but not by GNA11^Q209L^ with the loss of Pten or by the lack of oncogenes with the loss of Pten, using our established cells and melanoma cell lines. Increased migration was mainly regulated by the formation of lamellipodia through the induction of WRC by the loss of Pten and through EphA2 serine phosphorylation by the oncogenic MAPK pathway, suggesting an important role for the combination of oncogenes and tumor suppressors in cancer metastasis as well as tumor development.

The increase induced in migration ability by the loss of PTEN was only detected under the oncogenic MAPK pathway in cutaneous melanoma, but not under the GNA11^Q209L^ pathway in uveal melanoma (Fig. 2). Moreover, the combination of GNA11^Q209L^/PTEN loss was not beneficial for the metastasis or development of melanoma, and this is supported by epidemiological findings of their combination being absent in melanoma patients. Furthermore, although PTEN is a major tumor suppressor in cutaneous melanoma and the loss of PTEN is related to the metastasis of melanoma^8^, our established mouse cell lines suggest that the loss of Pten alone was not sufficient to induce the migration of melanoma (Fig. 2). The induction of WRC by the loss of Pten was not sufficient to induce cell migration in Pten^−/−^-melan-a and GNA11^Q209L^/Pten^−/−^-melan-a (Figs. 2, 4), suggesting the significance of serine 897 phosphorylation on EphA2 through oncogenic MAPK pathway. In consideration of the clinical implications of our results, the suppression of the oncogenic MAPK pathway by BRAF or MEK inhibitors may be a useful molecular targeted drug for cancer metastasis in BRAF^V600E^/PTEN loss melanoma. Moreover, similar to synthetic lethality or essentiality in cancer biology^5–7^, a more detailed understanding of genetic combinations may contribute to the development of novel therapeutic strategies for cancer metastasis.

The loss of PTEN mainly affects cancer development and cell proliferation by activating the PI3K-AKT pathway through the loss of its lipid phosphatase activity^28,29^. Although the loss of Pten activated the PI3K-AKT pathway in our established cells (Fig. 1), the PI3K-AKT pathway and EMT may be an unlikely mechanism increasing the migration of our cells (Supplementary Fig. S2). Therefore, we examined the increased expression of some WRC components following the loss of Pten (Fig. 4) through its protein phosphatase activity. Due to the higher frequency of loss of PTEN than the oncogenic *PIK3CA* mutation in melanoma, the significance of the loss of the protein phosphatase activity of PTEN is conceivable in melanoma malignancy.

The loss of PTEN and the oncogenic MAPK pathway are both required for the migration of melanoma in addition to the formation of lamellipodia. Since an immunoprecipitation assay showed that the inhibition of RSK did not affect the formation of WRC (data not shown), the oncogenic MAPK-EphA2 axis may play an important role either to activate WRC or localize WRC into lamellipodia. This concept is supported by previous findings showing that EphA2 activated WRC through RAC1-GEF ARHGEF16, also known as Ephexin4^30^. Nevertheless, the importance of WRC for the migration ability of our established cells was demonstrated (Figs. 3C, 4B). Furthermore, the clinical importance of WRC is supported by previous findings showing that the gain-of-function mutation of RAC1, which activates WRC, and also the gain-of-function mutation of PREX2, another RAC1-GEF, play an oncogenic role in melanoma^14,31^. In addition to their oncogenic roles, not only RAC1 but also EphA2 are involved in resistance to BRAF inhibition in melanoma^15,32^. These findings strongly support the significance of the oncogenic MAPK-EphA2 axis in melanoma malignancy.

Non-canonical EphA2 phosphorylation at Ser897 (pS-EphA2) has oncogenic and drug-resistant functions in solid cancers, including melanoma^32–34^. Consistent with previous findings of its phosphorylation being regulated by the serine/threonine kinase, AKT or RSK^17,35^, pS-EphA2 was mainly regulated through RSK and slightly through AKT in our established cells, which was observed in BRAF^V600E^ cells or Pten^−/−^ cells, respectively (Fig. 5A). Since Pten^−/−^ cells, which have active AKT (Fig. 1), did not show any increase in migration (Fig. 2A) and a PI3K inhibitor and AKT inhibitor did not suppress increases in the migration of BRAF^V600E^/Pten^−/−^ melan-a cells (Supplementary Fig. S2B), pS-EphA2 by AKT activity may be insufficient to induce cell migration. Alternatively, the present results support increases in migration ability and the formation of lamellipodia through the RSK-pS-EphA2 axis (Fig. 5). Although the mechanisms by which pS-EphA2 regulates the formation of lamellipodia have not yet been elucidated in detail, the results obtained from the inhibition of RSK or EphA2 (Fig. 5) suggest that the RSK-pS-EphA2 axis is required for the formation of lamellipodia. WRC is activated by EphA2 through RAC1 as described above^30,36^; therefore, WRC is an important intersection in melanoma migration from oncogenic BRAF signaling and Pten loss-mediated signaling.

We attempted to establish a model to examine the effects of genetic combinations using the mouse melanocytes, melan-a cells^20^. In comparisons with a genetically engineered mouse melanoma model, our model cell lines were simplified by excluding the effects of the tumor microenvironment, genetic backgrounds, and external mutagens, such as ultraviolet exposure, which have been shown to contribute to malignant phenotypes. The limitation of our established cell lines is that they were derived from a single cell clone. To overcome this issue, we confirmed consistent results in two alternative experiments using the knockdown of Pten in our established cells (Supplementary Fig. S1B) and human melanoma cell lines (Fig. 2C). These results support the loss of Pten inducing migration ability in cells with oncogenic MAPK activation.

In summary, the present study provides insights into the mechanisms underlying increases in cell migration by the loss of PTEN under the oncogenic MAPK pathway, which was mediated by the induction of some WRC subunits and through the serine phosphorylation of EphA2 by RSK. The present results also clarified the role of the combination of genetic alterations in cancer metastasis and will contribute to the development of novel therapeutic strategies for cancer metastasis.

## Materials and methods

### Plasmid preparation and lentivirus production

The plasmids used in the present study were pLenti CMV-BRAF V600E and pLenti-CMV-GNA11 Q209L, which subcloned human BRAF^V600E^ or GNA11^Q209L^ cDNA into pLenti CMV Hygro DEST (a gift from Eric Campeau & Paul Kaufman (Addgene plasmid #17454)^37^. LentiCas9-Blast was a gift from Feng Zhang (Addgene plasmid #52962; http://n2t.net/addgene:52962; RRID:Addgene_52962)^38^. Lentivirus particles were produced as previously described^39^.

The Arp2/3 inhibitor, CK-689, and the PI3K inhibitor, LY294002, were purchased from Merck KGaA (Darmstadt, Germany). The AKT inhibitor, MK-2206, was obtained from Active Biochemicals (Wan Chai, Hong Kong). The RSK inhibitors, BI-D1870 and LJH685, were supplied by BioVision (BioVision, Milpitas, CA, USA) and Cayman Chemical (Cayman Chemical, Ann Arbor, MI, USA), respectively. ALW II-41-27 was purchased from MedChemExpress (Monmouth Junction, NJ, USA).

### Cell cultures

The mouse melanocyte cell line, melan-a was kindly gifted from Dr. Bennett DC (St George’s, University of London, UK) and cultured as previously described^20^. Stable melan-a cells expressing spCas9 were established by the lentivirus infection of lentiCas9-Blast and referred to melan-a/Cas9. Next, stable melan-a/Cas9 cells expressing BRAF^V600E^ or GNA11^Q209L^ were established by the lentivirus infection of pLenti CMV-BRAF V600E or pLenti-CMV-GNA11 Q209L, selected and maintained with blasticidin S or hygromycin, and referred to BRAF^V600E^-melan-a and GNA11^Q209L^-melan-a, respectively. Pten sgRNA (524304_SGM, Thermo Fisher Scientific, Waltham, MA, USA) at a final concentration of 12.5 nM was transfected by Lipofectamine RNAiMAX reagent (Thermo Fisher Scientific). Pten^−/−^-melan-a, BRAF^V600E^/Pten^−/−^-melan-a, and GNA11^Q209L^/Pten^−/−^-melan-a cells were established after single cell cloning.

UACC257, Malme3M, and MP41 human melanoma cells were cultured in RPMI-1640 medium (Nissui, Tokyo, Japan) containing 10% fetal bovine serum (FBS) and penicillin/streptomycin/L-glutamine. MeWo and SK-MEL-2 human melanoma cells were cultured in Dulbecco’s Modified Eagle’s Medium (Nissui) containing 10% FBS and penicillin/streptomycin/L-glutamine.

### Cell growth assay

Cells were seeded at a density of 1 × 10^5^ cells/3.5 cm dish and were then collected at each day. Due to confluency, cells were transferred to 10 cm dish on Day4 and then collected at day 5, 6, and 7. The number of living cells was counted manually under a microscope.

### siRNA transfection

In siRNA knockdown experiments, siRNA for mouse Pten (s72351 and s72352, Thermo Fisher Scientific, Rockford, IL, USA), siRNA for human PTEN (s61222 and s61224, Thermo Fisher Scientific), siRNA for Abi1 (s61800 and s61801, Thermo Fisher Scientific), or negative control #1 (Thermo Fisher Scientific) was used for transfection at a final concentration of 12.5 nM in melanoma cells by Lipofectamine RNAiMAX reagent (Thermo Fisher Scientific) as previously described^40^.

### Western blotting analysis

Whole-cell extracts (10 µg/lane) were prepared as previously described^40^. Briefly, whole-cell lysates were collected in whole-cell lysis buffer (20 mM HEPES pH 7.6, 0.5 M NaCl, 1.5 mM MgCl_2_, 1 mM EDTA, 0.1% Triton X-100, and protease inhibitors (20 mM β-glycero-phosphate, 1 mM sodium orthovanadate, 1 mM phenyl-methylsulfonyl fluoride, 1 mM dithiothreitol, 10 mg/mL aprotinin, and 10 mg/mL leupeptin)). Regarding nuclear/cytoplasmic protein preparations, cells were lysed in lysis buffer C (20 mM HEPES pH 7.6, 0.04 M NaCl, 1.5 mM MgCl_2_, 1 mM EDTA, 0.1% Triton X-100, and protease inhibitors). After centrifugation, supernatants were collected as cytoplasmic extracts. Precipitates were again lysed in whole-cell lysis buffer and collected as nuclear extracts. Equal amounts of protein were resolved by electrophoresis on 7.5% or 10% acrylamide gels and transferred to an Immobilon-P nylon membrane (Millipore, Billerica, MA, USA). The primary antibodies used were FLAG (M2) from Signa-Aldrich (St. Louis, MO, USA), PTEN (#9188), Phospho-ERK1/2 (Thr-202/Tyr-204; #9101), Phospho-AKT (Ser-473; #9271), Abi1 (#39444), WAVE2 (#3659), CYFIP1 (#81221), EphA2 (#6997), Phospho-EphA2 (Ser-897; #6347), and Phospho-RSK (Ser-380; #11989) from Cell Signaling Technology (Beverly, MA, USA) and YAP (sc-15407), ERK1/2 (sc-514302), AKT (sc-1618), RSK1 (sc-231), and β-actin (sc-47778) from Santa Cruz Biotechnology (Santa Cruz, CA, USA). Antibodies were detected using horseradish peroxidase-conjugated anti-rabbit (P0448, DAKO, Glostrup, Denmark), anti-mouse (P0260, DAKO), and anti-goat IgG (P0449, DAKO), and visualized by the ECL system (GE Healthcare Bioscience, Piscataway, NJ, USA).

### Time-lapse microscopy

Cells were treated with inhibitors and continuously photographed every 5 min for 2 h in a CO_2_ incubator (37 °C, 5% CO_2_) using a time-lapse imaging system (Carl Zeiss Cell Observer). Images were acquired using an imaging system. The resulting images were processed by the Manual Tracking Plugin of ImageJ Fiji to track the number of cells. Accumulated distances in each cell were acquired using the Chemotaxis and Migration Tool (ibidi GmbH).

### Immunofluorescence

Cells were seeded on glass coverslips (Matsunami Glass, Osaka, Japan). Two days after seeding, cells were incubated with inhibitors and ligands or transfected with siRNAs. Cells were rinsed in cold PBS and fixed in 4% paraformaldehyde at room temperature for 25 min. After fixation, cells were permeabilized in PBS containing 0.5% Triton X-100 for 3 min and washed by PBS. Cells were incubated for 40 min with primary WAVE2 antibodies and then incubated with isotype-specific secondary antibodies conjugated with Alexa Fluor 488 and Rhodamine Phalloidin (Thermo Fisher Scientific) for 30 min. These antibodies were diluted in PBS containing 0.5% BSA. Microscopy was performed using a Zeiss LSM 700 confocal microscope (Oberkochen, Germany) and the cells with lamellipodia-like structure or the lamellipodia, which was double-stained with phalloidin and WAVE2, were counted.

### Statistical analysis

Significance was calculated using Graphpad Prism software (GraphPad Software, Inc., San Diego, CA, USA). More than three means were compared using a two- or one-way ANOVA with the Bonferroni correction, and two means were compared using the unpaired Student’s *t*-test. *p* < 0.01 was considered to be significant.

### Supplementary Information

Supplementary Figure S1.

## Data Availability

The datasets used and/or analysed during the current study available from the corresponding author on reasonable request.
