# Supplementary material for: Cooperative function of oncogenic MAPK signaling and the loss of Pten for melanoma migration through the formation of lamellipodia
Source: Sci Rep. 2024 Jan 17;14:1525. doi: 10.1038/s41598-024-52020-8 (PMC10794247; doi:10.1038/s41598-024-52020-8)
Supplement: Supplementary file 1 — Supplementary Figure S1. [file 41598_2024_52020_MOESM1_ESM.pdf]

Supplementary Figure S1

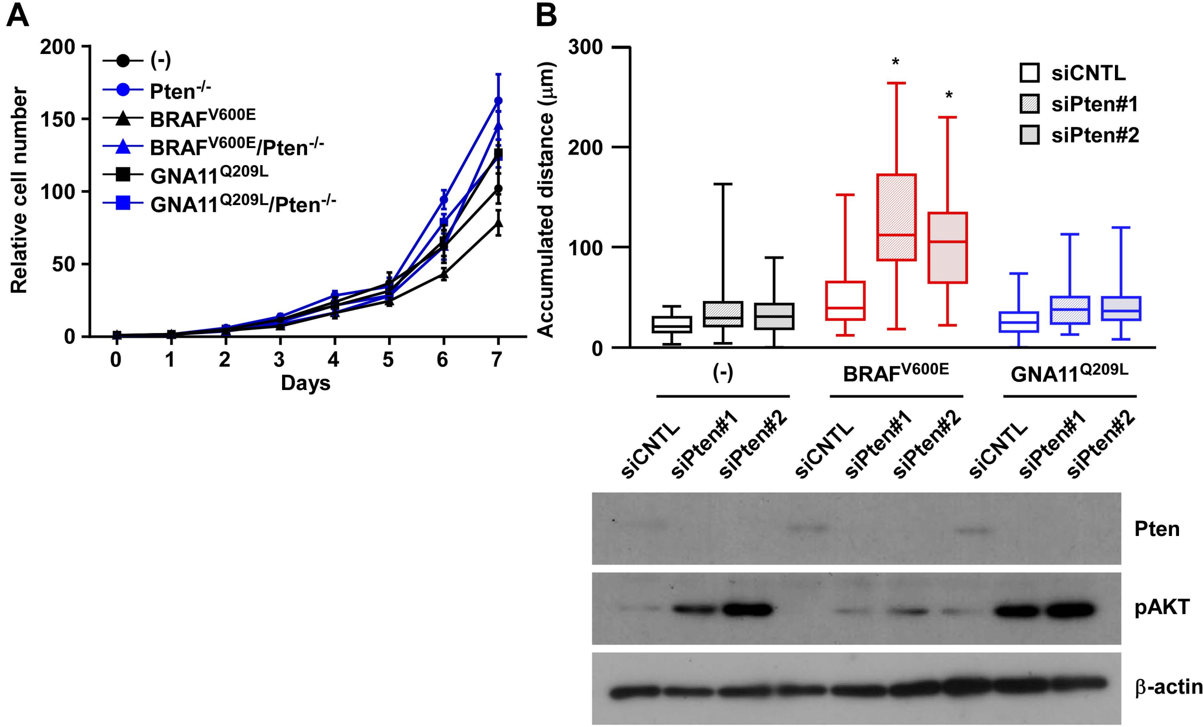

Supplemental Figure S1. $BRAF^{V600E}$ and the loss of Pten regulate cell migration, but not cell growth *in vitro*.

A Each cell was counted for 7days. Data are shown as the mean ± SD of at least three independent experiments. **B** Melan-a/Cas9, $BRAF^{V600E}$-melan-a/Cas9, or $GNA11^{Q209L}$-melan-a/Cas9 cells was transfected with siRNAs for Pten (#1 or #2). After 96 h, cell migration was observed using the time-lapse imaging system for 3 h (Upper) or whole-cell lysates were subjected to Western blotting (Lower). Other conditions were similar to those in Fig. 2A.

Supplementary Figure S2

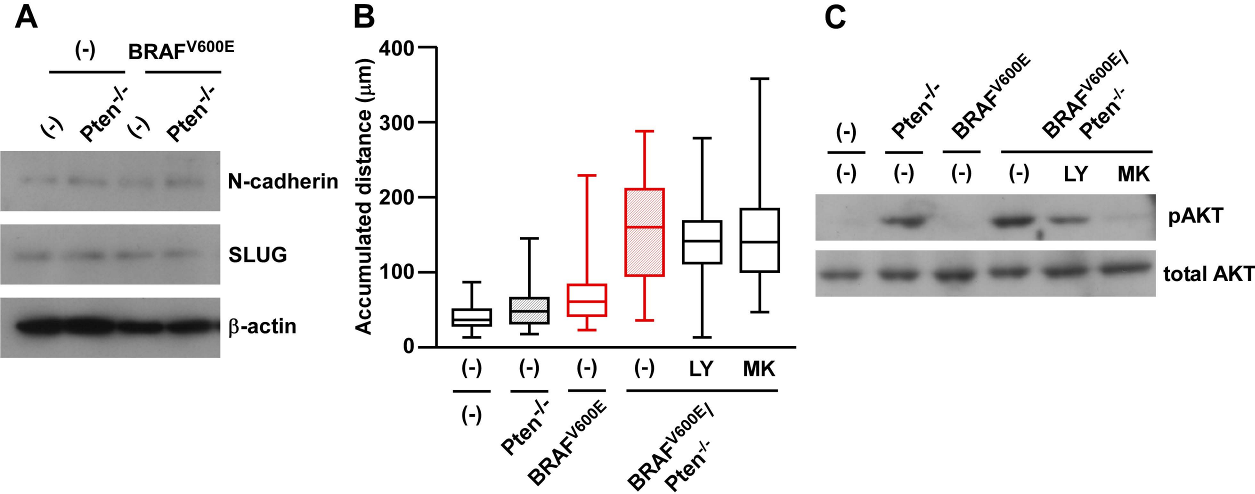

Supplemental Figure S2. Epithelial-to-mesenchymal transition or PI3K-AKT activation did not mediate the increased migration ability induced by the loss of Pten under the $BRAF^{V600E}$ background.

A, Whole-cell lysates from established melan-a cells were subjected to Western blotting. **B,** $BRAF^{V600E}/Pten^{-/-}$-melan-a/Cas9 cells were pre-treated with a PI3K inhibitor, 10 μM LY294002 (LY), or AKT inhibitor, 1 μM MK2206 (MK), for 24 h and cell migration was observed using the time-lapse imaging system for 3 h. Other conditions were similar to those in Fig. 2A. **C,** $BRAF^{V600E}/Pten^{-/-}$-melan-a/Cas9 cells were treated with 10 μM LY294002 (LY) or 1 μM MK2206 (MK) for 1 h and whole-cell lysates were subjected to Western blotting.

Supplemental Figure S3

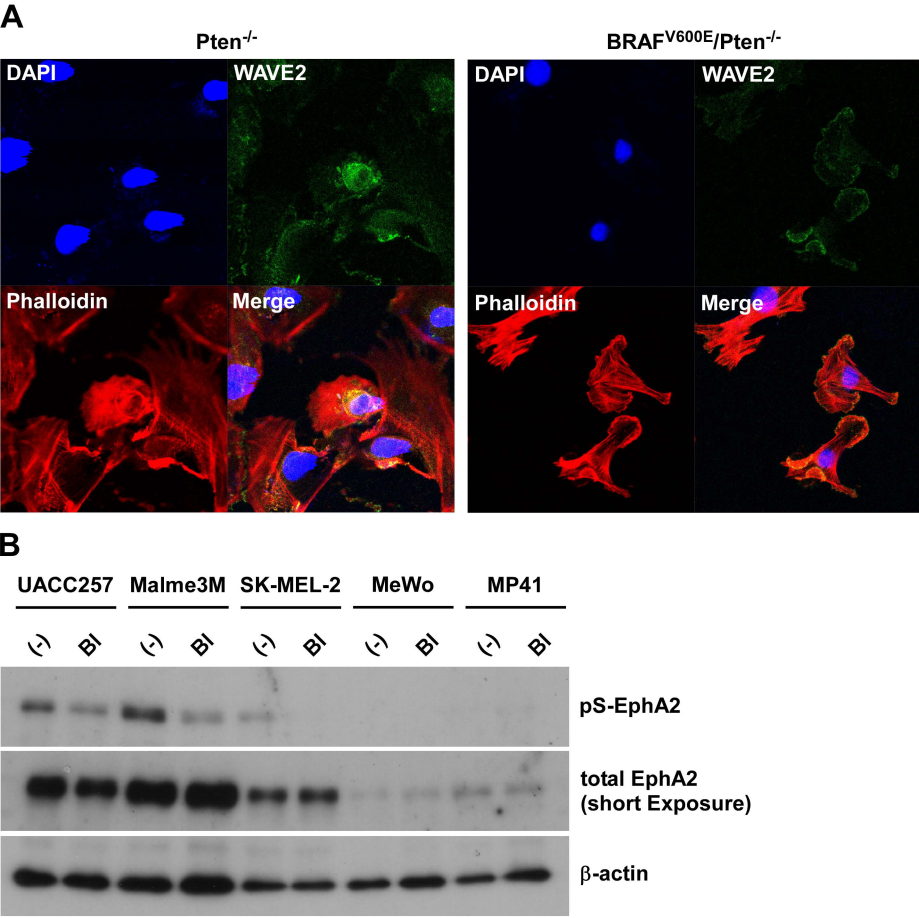

Supplemental Figure S3. Lamellipodia formation in BRAF^{V600E}/Pten$^{-/-}$-melan-a cells.

A, Pten$^{-/-}$-melan-a cells and BRAF^{V600E}/Pten$^{-/-}$-melan-a cells were stained with WAVE2 (green), rhodamine-conjugated phalloidin (red), and DAPI (blue). **B,** Human melanoma cells were treated with 10 μM BI-D1879 (BI) for 18 h and whole cell lysates were subjected to Western blotting.

Supplementary Figure S4

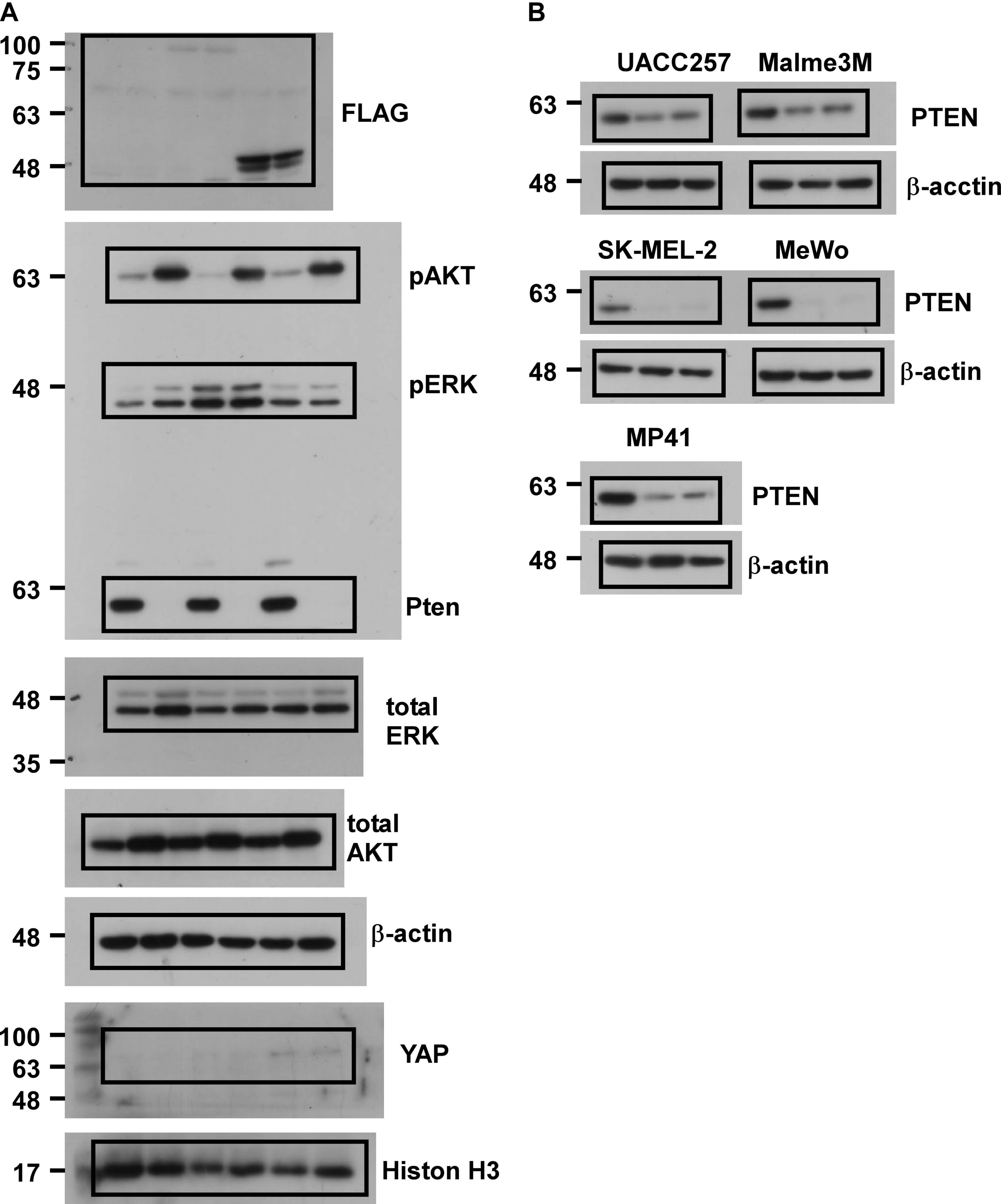

Supplementary Figure S4

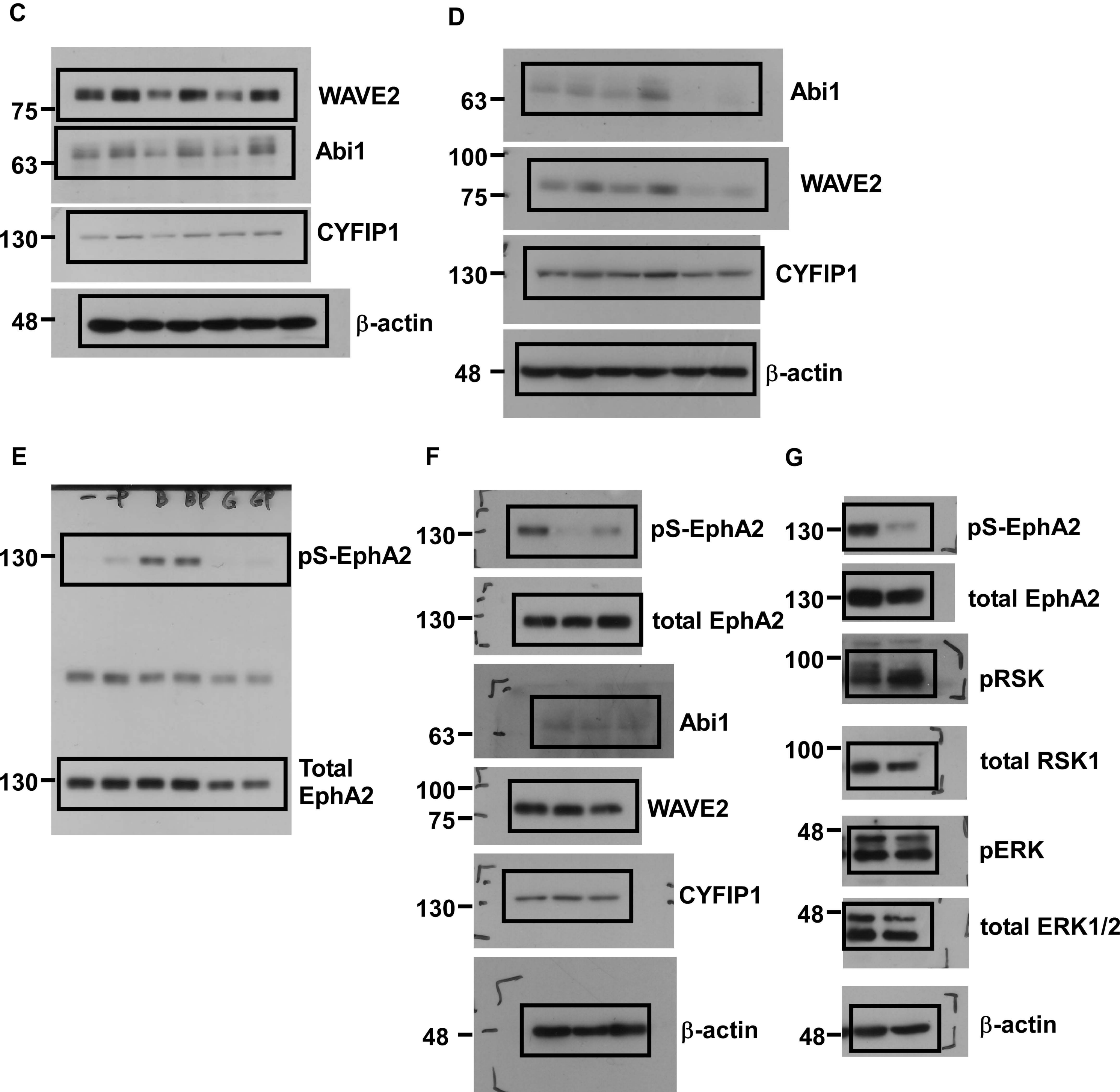

Supplementary Figure S4

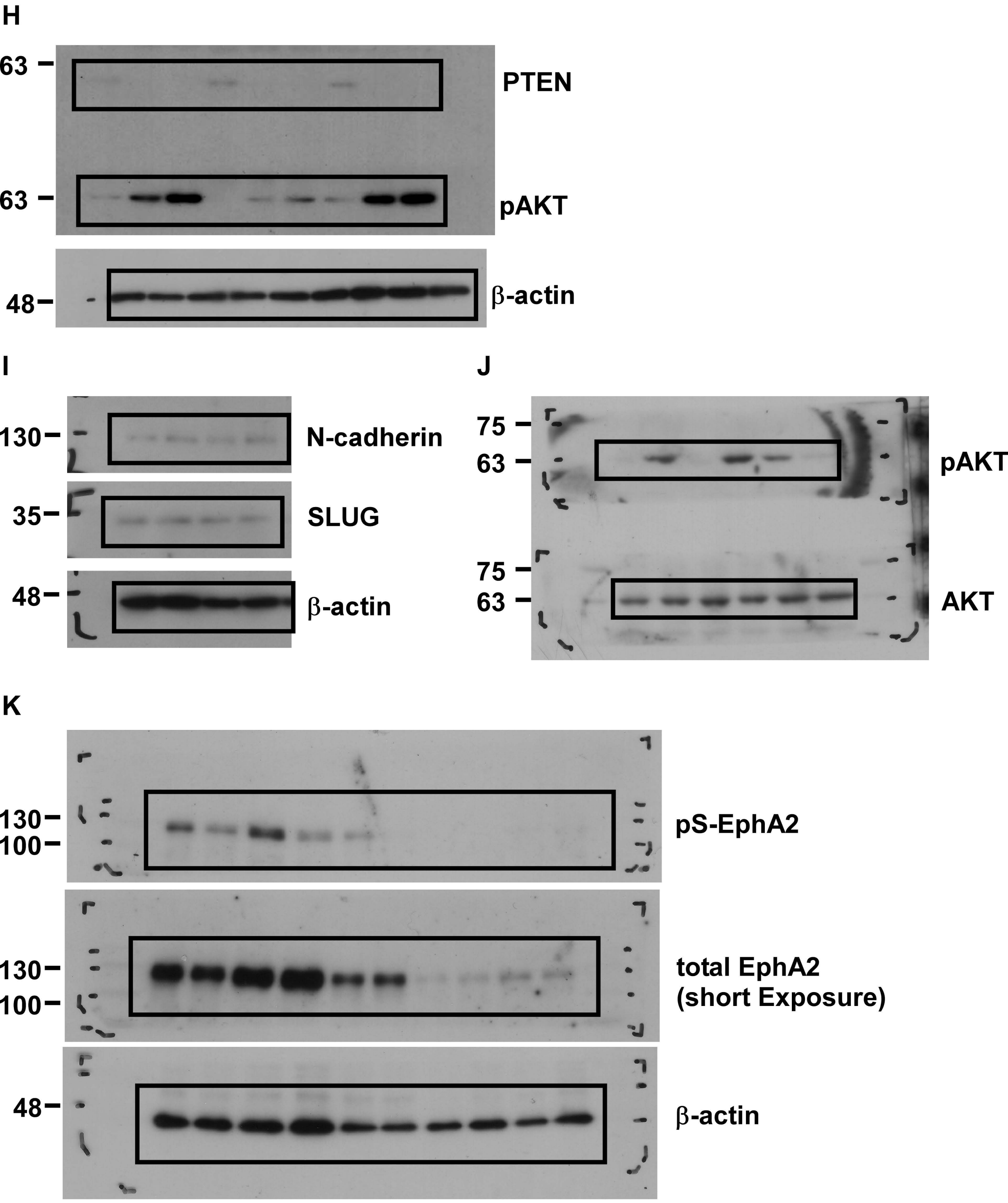

Supplementary Figure S4. Uncropped scans of all blots.
Uncropped scans of Figure 1 (A), Figure 2C (B), Figure 4A (C), Figure 4C (D), Figure 5A (E), Figure 5D (F), Figure 5G (G), Supplementary Figure S1B (H), Supplementary Figure S2A (I), Supplementary Figure S2C (J), and Supplementary Figure S3B (K) were shown.
